# Strategies to Promote Health System Strengthening and Global Health Security at the Subnational Level in a World Changed by COVID-19

**DOI:** 10.9745/GHSP-D-21-00478

**Published:** 2022-04-28

**Authors:** Martin Alilio, Neetu Hariharan, Elizabeth Lugten, Kama Garrison, Rhea Bright, Wilberforce Owembabazi, Uwem Inyang, Saad El-Din Hassan, Kelly Saldana

**Affiliations:** a Bureau for Global Health, U.S. Agency for International Development, Washington, DC, USA.; b U.S. Agency for International Development, Camris International, Bethesda, MD, USA.; c U.S. Agency for International Development, Credence LLC, Vienna, VA, USA.; d U.S. Agency for International Development, Social Solutions International, North Bethesda, MD, USA.; e Social Solutions International, North Bethesda, MD, USA.; f U.S. Agency for International Development Uganda, Kampala, Uganda.; g U.S. Agency for International Development Nigeria, Abuja. Nigeria.; h U.S. Agency for International Development Cambodia, Phnom Penh, Cambodia.

## Abstract

Structural weaknesses in national health systems have led to huge variations in responses to COVID-19. This calls for a unified approach to health security and essential health services as public health threats and the expectation for health care systems to provide improved access and services at affordable cost increases.

## Key Messages

- Achieving strong, resilient health systems that reach both global health security and universal health coverage goals requires a unified approach to how we work across the system.
- We propose the following 3 key overarching approaches:

- Strengthen comprehensive district planning (i.e., to be successful, district planners must understand how various public health programs come together within their communities, the range of stakeholders who have a role to play in health efforts, and how public and private funding and information systems can be leveraged at the local level to achieve their objectives).
- Support flexibility of district health systems to break the silos of vertical programs and take on new roles as circumstances warrant.
- Use the ongoing roll-out of COVID-19 interventions as an opportunity to address management, coordination, and integration challenges at the district level.

## INTRODUCTION: AN UNPRECEDENTED HEALTH CRISIS

The scale and severity of the global health and economic impacts caused by the coronavirus disease (COVID-19) pandemic remain difficult to estimate.^1^ However, it is clear from the subsequent COVID-19 waves experienced in India, South Africa, and other countries that the pandemic has placed tremendous pressure on health systems and created a catastrophic situation for millions of people in low- and middle-income countries (LMICs) worldwide. The response to the COVID-19 pandemic has shifted resources and attention toward tertiary care at the expense of other essential services.^2^ As such, many people living in LMICs no longer have access to routine preventive health and treatment services.

According to the World Health Organization (WHO), since the outbreak of COVID- 19 in March 2020, approximately 68 of 129 countries reported moderate to severe disruptions in vaccination campaigns, leaving more than 80 million children at risk.^3^ Infectious disease outbreaks often lead to a short-term breakdown of health systems, which then result in long-lasting reversals of public health gains.^4^ At the subnational level—which we will reference as district health systems hereafter—these consequences can be mitigated by improved resourcing, planning, and coordination of the areas that overlap between public health and health care (e.g., supply chain systems, financial management, public health campaigns, information systems, and community health infrastructure). However, the available literature on the response to COVID-19 continues to highlight the challenges involved in integrating these efforts across the health sector and points to inadequate resources and the relentless political pressure that forces countries to choose what parts of the health system receive new resources.^5^^–^^8^ Unfortunately, the literature offers few specific practical solutions on how these challenges might be addressed or actions that might be taken to strengthen the critical weak links that exist between these disparate functions at the district level. This is especially challenging due to the contextual differences between districts even within a country.

The literature offers few practical solutions on how health systems challenges might be addressed or actions that might be taken to strengthen the critical weak links that exist between disparate functions at the district level.

In this viewpoint, we seek to illustrate some of the critical health systems challenges at the district level and discuss problems associated with alignment, integration, and coordination. We offer potential solutions on how to align national and local priorities and policies to support countries to build health systems that are strong and resilient.

## STRENGTHENING HEALTH SYSTEMS TO ACHIEVE GLOBAL HEALTH SECURITY AND UNIVERSAL HEALTH COVERAGE GOALS

Before the COVID-19 pandemic, at least half of the people in the world did not receive the health services that they needed and approximately 100 million people were pushed into extreme poverty each year because of out-of-pocket spending on health care.^9^ COVID-19 exacerbated this situation as demand for acute care surged for people who were unable to pay for services due to loss of income and health insurance coverage.^10^ Countries were forced to make difficult decisions to balance the immediate demands of COVID-19 with ongoing strategic planning and coordinated action to maintain the delivery of essential health services, avoid system collapse, and safeguard against future events. Additional health system weaknesses such as inadequate infrastructure and supplies (e.g., limited beds, ventilators, and testing equipment) and extreme staff shortages (i.e., increased staff illnesses and deaths due to lack of personal protective equipment and appropriate infection prevention control) hindered the overall quality of care and forced physicians to decide who received care and who did not.

The experience of COVID-19 also demonstrated that even health care systems with high levels of service coverage and financial protection may not have been sufficiently resilient and may have had difficulties adapting and responding to major disruptions.^11^ Building health systems resilience capacities can help LMICs absorb, adapt to, and transform in the face of shocks and stressors to maintain delivery of quality essential health services outlined by universal health coverage (UHC) and global health security (GHS) goals. A good illustration of this capacity in action during COVID-19 is that some countries were able to direct potential COVID-19 patients to other facilities for treatment or use existing referral systems and digital technologies. For example, before 2019, with the support of international donors, Indonesia worked to strengthen the human resources needed to manage their health information system and ecosystem to provide real-time high-quality data for strategic use, such as the development of policy that addressed challenges to the health workforce. These investments proved to be crucial for supporting health care workers during COVID-19 and permitted the Indonesian Ministry of Health to undertake rapid development and deployment of data dashboards on the health workforce that facilitated decision making during response planning, which is a key element of adaptive capacity.^12^^,^^13^

Broadly, the global insecurity resulting from the COVID-19 pandemic and other public health emergencies highlighted yet again that strong and resilient health systems are at the heart of ensuring successful responses and effectively mitigating risks posed by recurring health crises (Box).^14^^–^^19^ GHS is an integral aspect of any health care system and is inextricably linked to UHC goals. Two examples of this are risk mitigation and the use of service statistics. Risk mitigation at the subnational level requires infrastructure and capabilities (e.g., laboratories, immunizations, and a well-equipped health workforce) that can support routine health service delivery through UHC, as well as fight existing epidemics, detect and respond to new outbreaks, and protect populations against public health emergencies. High-quality service statistics are important to UHC as they can be used to describe disease burden and epidemiology and are linked to the distribution of skilled human resources and funding. Understanding the patterns of service statistics is also critical to GHS. Whenever there is a deviation in trends, as in outbreaks, these statistics are useful data to support any notifiable and reportable event immediately through the appropriate channels, while also identifying the availability of local resources to help respond to or mitigate further spread of novel events. As countries strive to achieve both UHC and GHS goals, they need to determine how to bolster their health system capacity and improve performance to deliver better results and sustain efforts. By pursuing an integrated health system strengthening (HSS) approach, which encompasses strategies, responses, and activities designed to promote sustained improvements in health systems performance, countries are more likely to reach and maintain UHC and GHS goals.

BOX **Defining Key Terms**

**District**: The level where health policies and health sector reforms are interpreted and implemented. The World Health Organization Global Program Committee in 1986 adopted the following definition of the district health system^14^:

*A district health system based on primary health care is a more or less self-contained segment of the national health system. It comprises first and foremost a well-defined population, living within a clearly delineated administrative and geographical area, whether urban or rural. It includes all institutions and individuals providing health care in the district, whether governmental, social security, non-governmental, private, or traditional. A district health system, therefore, consists of a large variety of interrelated elements that contribute to health in homes, schools, workplaces, and communities, through the health and other related sectors.*

**Primary health care**: A whole-of-society approach to health that aims at ensuring the highest possible level of health and well-being and their equitable distribution by focusing on people's needs and as early as possible along the continuum from health promotion and disease prevention to treatment, rehabilitation, and palliative care, and as close as feasible to people's everyday environment.^15^

**Universal health coverage**: A global effort to ensure all people and all communities have access to preventive, curative, rehabilitative, and palliative health services that are of sufficient quality to be effective and do not result in financial hardship.^16^

**Global health security**: Focuses on strengthening systems that target prevention, detection, and response to infectious diseases wherever they occur in the world.^17^

**Resilient health systems**: Systems that are adaptive and have the capacity to transform, as necessary, to ensure the provision of accountable, affordable, accessible, and reliable care.^18^ A resilient health system can prevent, detect, and respond adequately to infectious disease threats. These systems are also able to adapt to everyday stressors, such as health worker absenteeism, and rearrange resources to avoid service disruptions and address immediate needs.

**One Health approach**: A collaborative, multisectoral, and transdisciplinary effort that addresses connections between people, animals, plants, and their shared environment.^19^

As countries strive to achieve both UHC and GHS goals, they need to determine how best to bolster their health system capacity and improve performance to deliver better results and sustain efforts.

## DISTRICT HEALTH SYSTEMS NEED URGENT REFORMS TO IMPROVE THEIR ABILITY TO PROVIDE BASIC HEALTH CARE AND COPE WITH OUTBREAKS

District health management teams (DHMTs) direct a vast array of complex operations that require coordination and effective integration of GHS and UHC activities into a comprehensive program of preventive, curative, rehabilitative care, and public health services. DHMTs are also responsible for implementing HSS initiatives. Yet, the public health efforts occurring within districts are often identified and implemented via siloed vertical programs that lack coordination with the rest of UHC activities.^20^ Understanding the dynamic, interactive elements of the health system, including those that are not explicit within the WHO building blocks (e.g., role of communities or surveillance), is key for DHMTs to successfully coordinate specific policies and initiatives and support integrated planning and implementation of programs at the subnational level.

For UHC and GHS initiatives to work in synergy and create an effective response to any public health threat, HSS programming at the district level must also include the implementation of governance and management structures for sharing resources and knowledge across public, private, and nonprofit sectors. For example, 6 years before the 2000 Ebola outbreak in Uganda involving 425 reported cases, Uganda initiated the process of decentralizing health care administration and placing primary authority at the district level to improve quality and increase utilization of health care services.^21^ This allowed the districts in Uganda to initiate comprehensive health planning and enabled district planners to understand how various public health programs came together within their communities. This deliberate planning also allowed a range of stakeholders who had a role to play in health efforts to come together; it enabled leveraging of public and private funding and information systems at the local level to achieve decentralized district objectives. The process helped to support the flexibility of district health systems to break the silos of vertical programs and take on new roles as circumstances warranted. As a result of the decentralization efforts, the district health system was adequately resourced and empowered to fulfill the necessary actions to contain the Ebola outbreak quickly and ensure minimal disruption to routine and essential service delivery. Additionally, DHMTs closely coordinated with the Ministry of Health to leverage technical capacity and resources from other ministries that might otherwise be perceived as outside their scope and mandate. This included collaborating with agriculture and environment ministries under the One Health approach (intersection of human health, animal health, and environmental health).^22^ As part of these critical reforms, Uganda also initiated the One Health Zoonotic Disease Prioritization process, which permitted districts to establish a transparent, unified forum with equal representation of government staff from One Health sectors actively involved in zoonotic disease prevention and control.^23^ In comparison, during the 2014 Ebola outbreak in Liberia, Sierra Leone, and Guinea, under-resourced district systems, limited authority, and minimal multisectoral coordination and collaboration ultimately resulted in the epidemic rapidly spiraling out of control.

Similarly, the COVID-19 pandemic demonstrated that countries with real-time integration of health security initiatives at the district level (e.g., Uganda, Cambodia, Laos, and Vietnam) maintained access to routine and essential health services.^24^^,^^25^ Hence, by identifying district-level pathways that promote integration, countries can take critical steps to ensure stronger and more resilient health systems that are able to effectively respond to future outbreaks.

By identifying district-level pathways that promote integration, countries can take critical steps to ensure stronger and more resilient health systems that are able to effectively respond to future outbreaks.

While the COVID-19 pandemic exposed major gaps in world health systems, the roll-out of accompanying interventions, including testing, patient care, and vaccines, represents an ongoing opportunity to strengthen health systems via a deliberate investment in public health and primary health care (PHC) at the subnational level. Most LMICs have a 3-tier system which includes the national, district, and community levels (Figure). The responsibility for health service delivery is shared across these levels by a variety of providers, including government, faith-based organizations, local and international nongovernmental organizations, and the private health sector.

**FIGURE:**
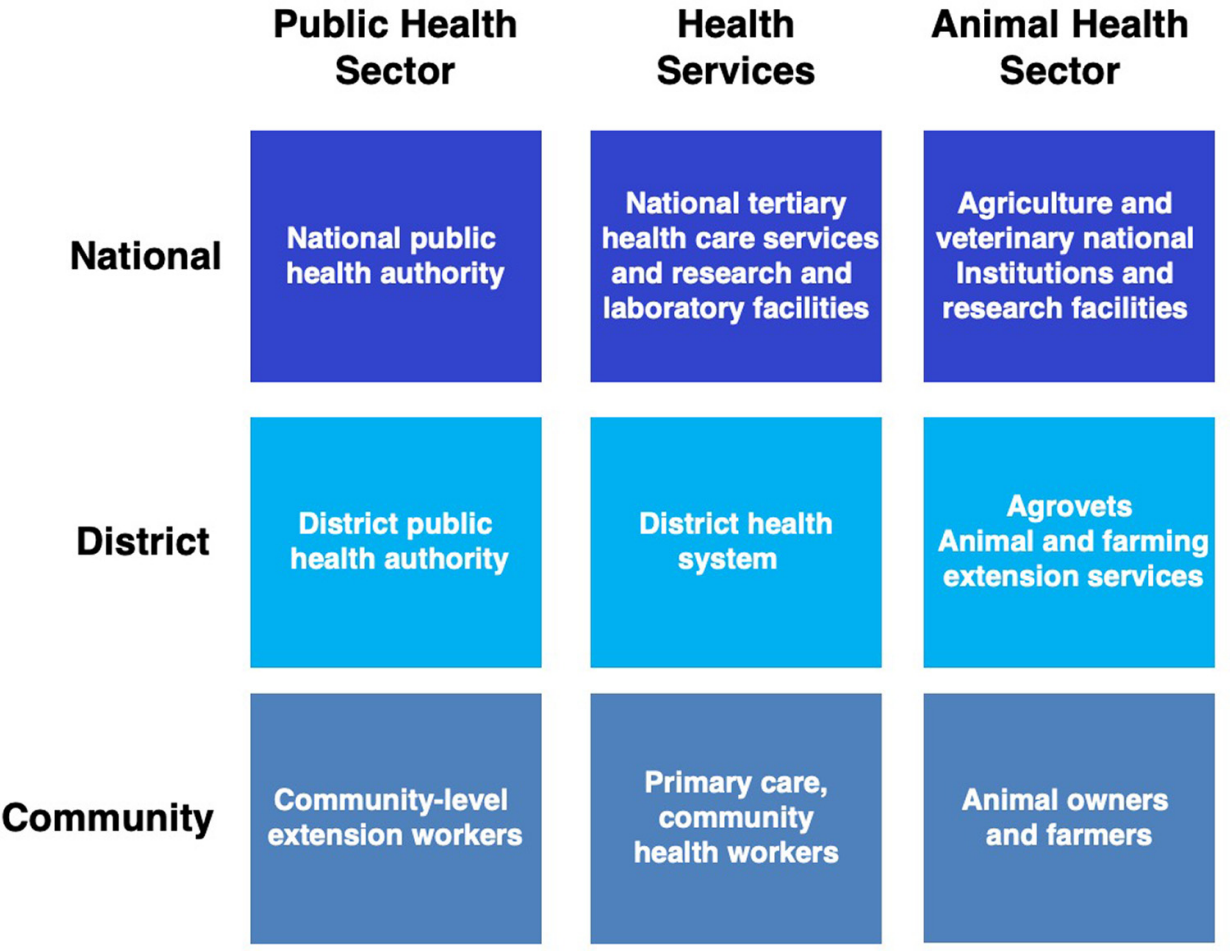
Health Systems Strengthening for Public Health: Core Levels and Functions

In most countries, some responsibilities of public health authorities fall outside of the Ministry of Health, requiring coordination with other ministries, including the ministries of agriculture and animal sectors, forestry, environment, education, defense, and transportation. Yet, there is still limited harmonization, resources, and capacity to bring these elements together at the district level. Additionally, under the auspices of national legislation, public health authorities are charged with executing essential public health functions. This leaves district health managers with limited authority to manage resources in ways that address inefficiencies and coordinate effectively across the system when they have responsibilities to coordinate across programs. Conflicts between the demands for central control and local discretion, limited institutional capacity, and political and cultural influences over their roles as district managers are common. Evaluations of district health systems in LMICs revealed that district health managers often feel powerless to address weaknesses in coordination because of the complicated matrix of accountability due to multiple cross-cutting flows of authority within and between the national and district levels and ever-changing political priorities.^26^^–^^28^ This is compounded by misalignment between public health institutes, national research facilities, national commissions such as those addressing HIV/AIDS, medical store departments, tertiary teaching hospitals, and the Ministry of Health, which becomes an acute problem during emergencies as subnational PHC tends to be limited to the delivery of clinical services.

## POTENTIAL SOLUTIONS TO ALIGN NATIONAL AND DISTRICT-LEVEL PRIORITIES TO BUILD A RESILIENT HEALTH SYSTEM

To achieve the goals of strong, resilient health systems that incorporate both GHS and UHC, we propose the following recommendations:

### 1. Strengthen Investments in Cross-Cutting Aspects of Health Systems That Promote Public Health at the Subnational Level

LMICs will need assistance in the form of cross-cutting health systems strengthening investment and technical support to integrate essential public health functions into existing subnational and PHC systems to achieve GHS and UHC goals. These investments will be particularly important in the overall efforts to decentralize health systems, as these have become increasingly complex operations that need to be carried out at the lowermost levels. GHS and UHC need to be addressed jointly to improve the coordination of efforts directed at the district level and could benefit from a more explicit review aimed at identifying critical interdependencies based on an analysis of system dynamics (Table 1). The Thirteenth General Program of Work released by WHO in 2018— embraced by most member LMICs — clearly states that GHS and UHC programs will require implementation that is jointly reinforcing. This will require ongoing attention to contextual issues, governance, and power structures, as well as identifying and addressing gaps in implementation.

**TABLE 1.** HSS Strategies to Integrate GHS and UHC

Explicit governance, organizational, and management strategies to promote the integration of GHS and UHC at the subnational level	Align national and local priorities and policies to ensure that GHS remains a critical component of PHC priorities and strategies.
	Integrate the supply chain system with a focus on forecasting, transportation, storage, and stock management to promote efficiency, good stewardship of donated commodities, and limit waste.
	Strengthen cold chain supply systems.
	Integrate public health campaigns, including vaccinations, mass drug administration, and bed net distribution to ensure they are a planned aspect of district health program management rather than separate donor-driven efforts.
Flexible finance systems that consider exceptional emergencies	Develop a transparent process for using local funds for emergency/outbreak response.
	Strengthen subnational budget oversight and encourage the use of transparent financial management systems.
Coordination to institute clear lines of communication	Generate clear definitions of shared cross-cutting objectives and aligned leadership roles.
	Engage the community and multiple stakeholders in defining and addressing population health needs.
	Develop and implement plans for shared infrastructure that included international points of entry and the cross-border districts.
	Reboot mechanisms that promote sharing and collaborative use of data and analytics.
	Create an explicit platform for donor coordination at the subnational level with clear links to government systems and local partners as applicable.
Integration of programs and institutions at the subnational level into a comprehensive range of preventive, curative, and rehabilitative health activities	Establish a senior-level position within the DHMT to coordinate HSS and GHS strategies.
	Strengthen the planning and management capacity of the DHMT with a focus on the mandate and the ability to integrate programs and institutions into a single, comprehensive district health system.
	Leverage resources from private-sector providers, including memoranda of understanding and public-private partnerships at district, facility, and community levels.
	Provide community health care workers with coordinated roles in pandemic prevention and response.

Abbreviations: DHMT, district health management team; GHS, global health security; HSS, health system strengthening; PHC, primary health care; UHC, universal health coverage.

Disruptions resulting from the COVID-19 pandemic have created an opportunity to integrate these efforts across existing programs used to deliver public health services. In Ghana, for example, based on a pilot study that used a seasonal malaria chemoprevention campaign to undertake TB and malnutrition screening among children aged 15 years and younger, the Ministry of Health is currently working to incorporate chemoprevention campaigns to prevent seasonal malaria together with TB and severe malnutrition screening.^29^ If carried forward in the future, such integration has the potential to change the way critical services are delivered and eliminate current system inefficiencies. Advanced coordination across health programming efforts with a focus on providing an integrated response to anticipated and unanticipated shocks and stressors will facilitate more rapid and effective responses in the future.

Advanced coordination across health programming efforts with a focus on providing an integrated response to anticipated and unanticipated shocks and stressors will facilitate more rapid and effective responses in the future.

Drawing lessons from COVID-19, special attention is also needed in building the capacity of DHMTs to coordinate and manage international emergency donations. Several countries received donated ventilators, oxygen concentrators, vaccines, and other commodities. Due to differences in specifications, site requirements, and supply chain system and operational challenges, these donations sometimes did not achieve their intended objectives and even constituted an added burden to the district health care system. Moreover, some donations such as those related to oxygen supply require a longer-term plan to ensure infrastructure and systems are in place for proper use and management.^30^ Strengthening supply chain systems to improve the process of equipment donation to and at the district level and establishing longer-term plans for certain donations will be necessary for the mutual benefit of donors and recipients alike.

Lastly, districts with an international point of entry play a pivotal role in controlling disease outbreaks. A closer look at the Ebola outbreak in West Africa reveals that the outbreak was for the most part concentrated in the cross-border districts of 3 Mano River Union countries of Sierra Leone, Guinea, and Liberia. There were no operational district health systems in place in these rural areas traversing 3 countries. Lack of capacity and coordination in these districts limited the ability to effectively respond to the Ebola crisis and there was no capacity to predict the outbreak. This is a major challenge for most LMICs, and the problem is rarely appreciated or addressed. At the onset of COVID-19, for example, point-of-entry districts lacked the necessary medical supplies (test kits and protective and laboratory equipment) and related information and communications equipment and support (e.g., mobile phones, computers, adapted software, data platforms, and internet), which profoundly limited the ability of several countries to contain transmission in border-crossing districts.^31^

### 2. Support the Flexibility of District Health Systems in Their Efforts to Accommodate New Roles as Circumstances Warrant

Collectively, building resilience capacity depends less on the focus of individual districts and more on how different mechanisms at the district level are integrated to work together. It also requires a holistic approach to HSS, including both health care services and public health functions. The implementation of GHS activities at the subnational level remains under the direct control of national-level institutions, such as presidential commissions. Emergency responses are organized under a task-force reactive model, and often efforts are not fully integrated into the ongoing health service provision system. LMICs urgently need to build capacities at the district level to fully manage public health emergency responses and to facilitate early detection of infectious disease outbreaks. This capacity is also vital for DHMTs to be able to lead efforts to detect and respond to changes in health system performance or community health status.

LMICs urgently need to build capacities at the district level to fully manage public health emergency responses and to facilitate early detection of infectious disease outbreaks.

We present several strategies for investment that can be used to create synergy between sectors at the subnational level (Table 2).^32^^–^^35^ These suggestions also ensure an agile workforce that can fully embrace core responsibilities, including their roles in cross-cutting aspects of health systems.

**TABLE 2.** Actions to Build Capacity, Establish Processes, and Promote Synergy at the Subnational Level

Create capacity within district health management teams to include all health professionals in one comprehensive system with management and deployment coordinated, as necessary	Promote the capacity for rapid response with teams capable of investigating and responding to outbreaks.
	Strengthen systems that promote strategic production, institutionalization, (re)deployment, and retention of human resources in alignment with needs that are defined at both national and subnational levels.
	Facilitate and implement models that integrate public health and health systems for optimal leverage and strategic use of human resources.
	Institute structures that sustain and advance one-health technical capacities as well as those that strengthen programs at academic and training institutions.
	Empower district and regional managers to promote transparency concerning workloads and planning among all health professionals regardless of current supervisory or management structures.
Build sustainable capacity to develop and implement laws, regulations, and policies within each country	Strengthen links between the community, civil society, and the private sector to increase transparency and accountability.
	Establish a participatory process that deliberately includes and empowers subnational authorities in the policy development and implementation process.
Support a culture of evidence-based decision making, collaboration, and improved information sharing	Support strategic improvements in methods used to integrate health information systems including relevant health surveillance data.
	Strengthen reporting systems and establish agreements, protocols, processes, and regulations that govern reporting at the country, regional, and global levels.
Institute robust facility-level protocols and programs to prevent and control infections	Support regular training and review of protocols in all health-related facilities.
	Combat antimicrobial resistance by supporting the translation of national rational use guidelines for medicines to subnational level actions.
	Address human and animal health, food production, and environmental aspects through multisectoral engagement and collaboration.

Moreover, countries need to also consider the roles played by additional stakeholders within the system, including the private sector, the nonprofit sector, and communities who are critical to ensuring health systems can prevent, detect, and respond to infectious disease threats and day-to-day disruptions. The objectives focused on creating this synergy at the subnational level must be clear and explicit. Rectifying these shortcomings as part of a successful and ongoing response to the COVID-19 pandemic, including equitable access to vaccines, will depend on efforts to strengthen the subnational health system that are focused on improving its capacity to integrate and coordinate critical health care initiatives.^36^

### 3. Use the Roll-Out of COVID-19 Interventions as Opportunities to Address Existing Challenges

With the roll-out of interventions directed at controlling the spread and treatment of COVID-19, the use of innovative approaches for facility and community-based patient care and vaccines provides a significant opportunity to address coordination issues at the subnational level. Specifically, these approaches might leverage in-house capacity and experiences from other clusters. For example, assets provided by the polio eradication program that promote sustainability and early mobilization of both human and financial resources might be used to initiate readiness activities in priority countries. As part of the COVID-19 response, a laboratory diagnostic network has been established that covers nearly all African countries with tools to detect severe acute respiratory syndrome coronavirus type-2 (SARS-CoV-2) using real-time polymerase chain reaction technology. By leveraging HSS efforts, these countries can build on this capacity to address a larger range of diseases and disease outbreaks. These investments provide an opportunity to address other issues, including supply chain weaknesses. While supply chain mechanisms associated with COVID-19 diagnostics have improved considerably since the beginning of the pandemic, they remain insufficient and highly vulnerable to potential industrial and transport shutdowns as well as export and border restrictions that may result from continued high worldwide demand. Moreover, challenges remain regarding the ongoing treatment of severe cases, including lack of essential equipment and human resources, even in countries with optimal health care systems.

Current immunization programs in LMICs focus on children and women of childbearing age. Efforts to vaccinate the entire population against COVID-19 may require combining with or leveraging other health campaigns to optimize the use of resources to meet the demand. DHMTs may also consider opportunities to formally link communities to the health system (e.g., social media listening, rumor monitoring, and response systems) to understand and address issues related to vaccine access, demand, confidence, and misinformation. Ongoing vaccination efforts will also require further coordination of the supply chain, human resources, information, and financing arrangements as components of the PHC system. This is also an area where international collaboration is vital to help tackle global vaccine access challenges. Almost 9 in 10 respondents to a recent United Nations global survey believe that international organizations (e.g., U.S. Centers for Disease Prevention and Control; WHO; GAVI Vaccine Alliance; United Kingdom's Foreign, Commonwealth, and Development Office; U.S. Agency for International Development; and Bill & Melinda Gates Foundation) will continue to be instrumental in helping stakeholders to achieve common goals and promote better preparedness at global, national, and subnational levels.^37^ Such collaboration should help define the scopes of engagement and roadmap for vaccine-related efforts at the local level from sharing health data to solving supply chain issues.

Failure to coordinate and integrate these efforts will represent a missed opportunity and will demonstrate that lessons highlighted by the COVID-19 pandemic have gone unrecognized. The capacity to deliver routine immunizations and conduct regular testing over a wide coverage area that reaches vulnerable populations would provide an excellent example of the integration of GHS and UHC. Missing this opportunity would severely limit the health system's ability to bounce back effectively and build stronger systems in the future.

The capacity to deliver routine immunizations and conduct regular testing over a wide coverage area that reaches vulnerable populations would provide an excellent example of the integration of GHS and UHC.

## REMAINING EVIDENCE GAPS: HOW TO IMPROVE SYNERGY BETWEEN HSS AND GHS IN LMICS

With the recent series of outbreaks globally, how countries reach UHC and GHS goals has evolved rapidly. Future investments toward HSS may also require robust and updated evidence on strategies to facilitate the integration of UHC and GHS initiatives in a way that would result in improved health outcomes and greater efficiencies. Examples include identifying and examining which and how various health care and public health services, programs, and functions may be combined or better harmonized; gathering more evidence on the effectiveness of strategies that facilitate the integration of services for greater efficiency and improved health outcomes; and exploring transformative management strategies to enable an agile UHC and GHS workforce that can deliver high-quality services. Some strategies have already been tested at the country level, for example, changes in payment systems, supervision, decentralization, community participation, and accountability mechanisms.^38^^,^^39^ Although some findings support the benefits of these strategies, there is little to no information available as to how they might be implemented both effectively and sustainably.

Similarly, research on strategies for community engagement during the global COVID-19 pandemic has been marred by unprecedented episodes of misinformation and uncertainty.^40^ This problem has limited awareness, access to and utilization of health services, and provision of appropriate services at the community level. Many public health programs, including disease-specific control measures, have adopted community engagement strategies to improve access and expand coverage. The increased use of community-based approaches may serve to enhance ownership in PHC and increase access to and utilization of health services by all for both communicable and noncommunicable diseases. Nonetheless, there remain great gaps in our knowledge as to how to understand and evaluate implementation strategies used for these approaches, including, but not limited to, innovative health care strategies at the interface of peripheral health services and communities, community capacity to sustain human and financial resource requirements, and how these strategies can be deployed for optimal responses to global pandemics.

Further research and learning efforts can bring insights into these issues and will certainly generate evidence on how to improve UHC and GHS programs as well as how to scale up and sustain community-based efforts and existing interventions. The proposed efforts should seek to adopt a systems-based perspective. This may require multidisciplinary research and learning teams and engaging multiple stakeholders including beneficiaries of the health system. Some key questions include:

- How can numerous parallel community activities be integrated into one sustainable community-based health package? The aim is to devise a system that efficiently and effectively addresses the key priorities identified in different regions. In other words, the package needs to be adapted to subnational priorities and should have built-in flexibility and adaptability so that its components can respond to changes in these priorities.
- How can different stakeholders at the subnational level (e.g., health system managers, health workforce members, and private sector institutions and communities) be drawn together to advise policy makers on how to ensure that HSS addresses GHS and UHC needs?

## CONCLUSION

Urgent support is needed at the subnational level of LMICs to integrate isolated programs and to facilitate the appropriate distribution of resources to ensure better integration of HSS and health security to advance progress toward UHC. A prime example of this priority is the COVID-19 interventions roll-out. We recommend a system analysis-based approach to increase resilience and improve GHS programs. Increased investment in improved technical and management capabilities will also be needed at the subnational level to address persistent problems and to ensure optimal functioning of the health system. Currently, there is little to no information available to inform the strategies discussed in this viewpoint. Implementation of these recommendations should include a research agenda aimed at understanding how to integrate parallel activities into one sustainable community-based health system that addresses key priorities and ensures that new directions are flexible and capable of responding to change. Implementation might also focus on how the various stakeholders might be drawn together to advise policy makers on the optimal strategies to be used to address current GHS and HSS needs at the subnational level. Despite the challenges associated with limited resources and lack of concrete evidence, there are several advantages to coordinating efforts at the subnational level, including geographical proximity, personal familiarity among the stakeholders, and a local context for the desired actions.

## Notes

**Peer Reviewed**
